# Avian influenza surveillance in domestic waterfowl and environment of live bird markets in Bangladesh, 2007–2012

**DOI:** 10.1038/s41598-018-27515-w

**Published:** 2018-06-20

**Authors:** Salah Uddin Khan, Emily S. Gurley, Nancy Gerloff, Md Z. Rahman, Natosha Simpson, Mustafizur Rahman, Najmul Haider, Sukanta Chowdhury, Amanda Balish, Rashid Uz Zaman, Sharifa Nasreen, Bidhan Chandra Das, Eduardo Azziz-Baumgartner, Katharine Sturm-Ramirez, C. Todd Davis, Ruben O. Donis, Stephen P. Luby

**Affiliations:** 1 icddr,b (International Centre for Diarrhoeal Disease Research, Bangladesh), Dhaka, Bangladesh; 2 Department of Population Medicine, University of Guelph, Guelph, Canada; 3 Centers for Disease Control and Prevention, (CDC), Atlanta, GA USA; 4 Department of Livestock Services, Ministry of Fisheries and Livestock, Dhaka, Bangladesh; 5 Biomedical Advanced Research and Development Authority, Washington D.C., USA; 6 Stanford University, Stanford, California, USA

## Abstract

Avian influenza viruses, including highly pathogenic strains, pose severe economic, animal and public health concerns. We implemented live bird market surveillance in Bangladesh to identify the subtypes of avian influenza A viruses in domestic waterfowl and market environments. We collected waterfowl samples monthly from 4 rural sites from 2007 to 2012 and environmental samples from 4 rural and 16 urban sites from 2009 to 2012. Samples were tested through real-time RT-PCR, virus culture, and sequencing to detect and characterize avian influenza A viruses. Among 4,308 waterfowl tested, 191 (4.4%) were positive for avian influenza A virus, including 74 (1.9%) avian influenza A/H5 subtype. The majority (99%, n = 73) of the influenza A/H5-positive samples were from healthy appearing waterfowl. Multiple subtypes, including H1N1, H1N3, H3N2, H3N6, H3N8, H4N1, H4N2, H4N6, H5N1 (clades 2.2.2, 2.3.2.1a, 2.3.4.2), H5N2, H6N1, H7N9, H9N2, H11N2 and H11N3, H11N6 were detected in waterfowl and environmental samples. Environmental samples tested positive for influenza A viruses throughout the year. Avian influenza viruses, including H5N1 and H9N2 subtypes were also identified in backyard and small-scale raised poultry. Live bird markets could be high-risk sites for harboring the viruses and have the potential to infect naive birds and humans exposed to them.

## Introduction

Highly pathogenic avian influenza (HPAI) A(H5N1) virus has been identified in domestic poultry and humans in Asia since 1997^1,2^. Bangladesh first reported HPAI in domestic poultry in March 2007^3^. From 2007 to 2013, the country reported 547 H5N1 outbreaks in domestic poultry in 51 (80%) of its districts^3^. Eighty-five percent of outbreaks were reported among poultry raised in commercial farms^4^. Eight human cases of H5N1, including one fatality, were reported from 2008 to 2015 after exposure to ill poultry purchased in live bird markets (LBM)^5^.

The steady supply of poultry in the LBM provides susceptible avian hosts for the avian influenza A viruses and may serve as a point for spreading infection between poultry^6^. Several locations in Asia and the Middle-East including Hong Kong, China, Indonesia, South Korea, Vietnam, Thailand, and Egypt have identified multiple subtypes of influenza viruses, including H5N1, in poultry in live-bird markets^7–11^.

In Bangladesh, live-bird markets are the primary hub for poultry marketing throughout the country^12,13^. Chickens, ducks, and geese are mostly traded alive because of cultural and religious preference for consuming freshly slaughtered poultry and the lack of refrigeration, particularly among rural households^13^. Local assembly traders purchase poultry from backyard and small producers in peripheral rural markets and sell them to distributors. Poultry are then transported to poultry wholesalers in districts or cities, where they are, in turn, purchased by poultry vendors. This process of poultry trading from purchase at the farm to purchase by the consumer usually takes 1–2 days^13^. At district and city markets, multiple species of birds were sold daily, and the vendors keep the birds until they are sold, creating opportunities for mixing unsold poultry with the fresh batch brought to the markets^14^.

Waterfowl brought in to live-bird markets are particularly important in the transmission pathway among domestic poultry because, once infected, they may shed influenza viruses for days through fecal and respiratory droppings usually without showing clinical signs of disease^15–17^. In contrast, mortality due to HPAI in the order *Galliformes*, which includes chickens and quail, is very high; they often show severe respiratory distress within 24 hours of infection and die within 2–3 days^18^. To determine which avian influenza A viruses were circulating among domestic poultry in Bangladesh, we conducted surveillance in domestic waterfowl brought to the live-bird markets.

## Results

During August 2007 and December 2012, we tested swab samples from 4,308 domestic waterfowl from four live bird markets. Most (86%) were adult waterfowl and 96% appeared healthy (Table 1). Most (93%) had been raised in backyards, and the remainder were from free ranging small-scale commercial flocks (Table 1). The average backyard flock had 18 birds (95% CI: 18–20), and the average small scale and commercial free ranging flock had 325 birds (95% CI: 293–358). Two hundred fifty (6%) of the sampled birds raised in backyards and 63 (19%) of the sampled birds raised as free ranging waterfowl flocks had at least one death from illness in their flock in the week preceding sampling. The poultry mortality ratio between the backyard and free ranging waterfowl were not significantly different (average mortality in backyard poultry was 1.2% vs. 0.9% among small-scale free ranging poultry, p = 0.07) (Table 1).

**Table 1:** Numbers of laboratory confirmed influenza A and influenza A/H5 waterfowl samples identified in the four rural live-bird markets, Bangladesh, 2007–2012.

Type of birds sampled	Number (N = 4308)	Influenza A – all subtypes (%, 95% CI)	Influenza A/H5 (%, 95% CI)
Ducks	3800 (88.2%)	186 (4.9%, 4.2–5.7)	71 (1.9%, 1.5–2.4)
Geese	508 (11.8%)	5 (1.0%, 0.3–2.2)	3 (0.6%, 1.2–17.3)
**Age group of the bird**			
Juvenile	610 (14.2%)	22 (3.6%)	6 (1.0%)
Adult	3698 (85.8%)	169 (4.6%)	68 (1.8%)
**Health status during sampling**			
Apparently healthy	4124 (95.7%)	188 (4.6%)	73 (1.8%)
Sick or dead*	184 (4.3%)	3 (1.6%)	1 (0.5%)
**Type of swab specimen**			
Oropharyngeal	705 (16.4%)	3 (0.4%)	1 (0.1%)
Cloacal	3354 (77.8%)	176 (5.3%)	72 (2.2%)
Fecal	249 (5.8%)	12 (4.8%)	1 (0.4%)
**Poultry flock size**	**Mean (95% CI)**	**n (%, 95% CI)**	**n (%, 95% CI)**
Backyard (n = 3991)	18.9 [17.6–20.1]	172 (4.3%, 3.7–5.0)	74 (1.9%, 1.4–2.3)
Small scale (n = 317)	325 [292.5–358.1]	19 (5.9%, 3.6–9.4)	0 (0%. −)
**Poultry mortality in the preceding week of sampling**	**Mean (95% CI)**	**Mean (95% CI)**	**Mean (95% CI)**
Backyard	1.2% [1.2–1.3%]	2.1% [1.9–2.3%]	1.1% [0.9–1.3%]
Small scale	0.9% [0.8–1.0%]	0.2% [0.03–0.5%]	None

Note: CI – Confidence Intervals.

*Seven dead waterfowl were sampled, none tested positive for influenza A viruses.

**Figure 1:**
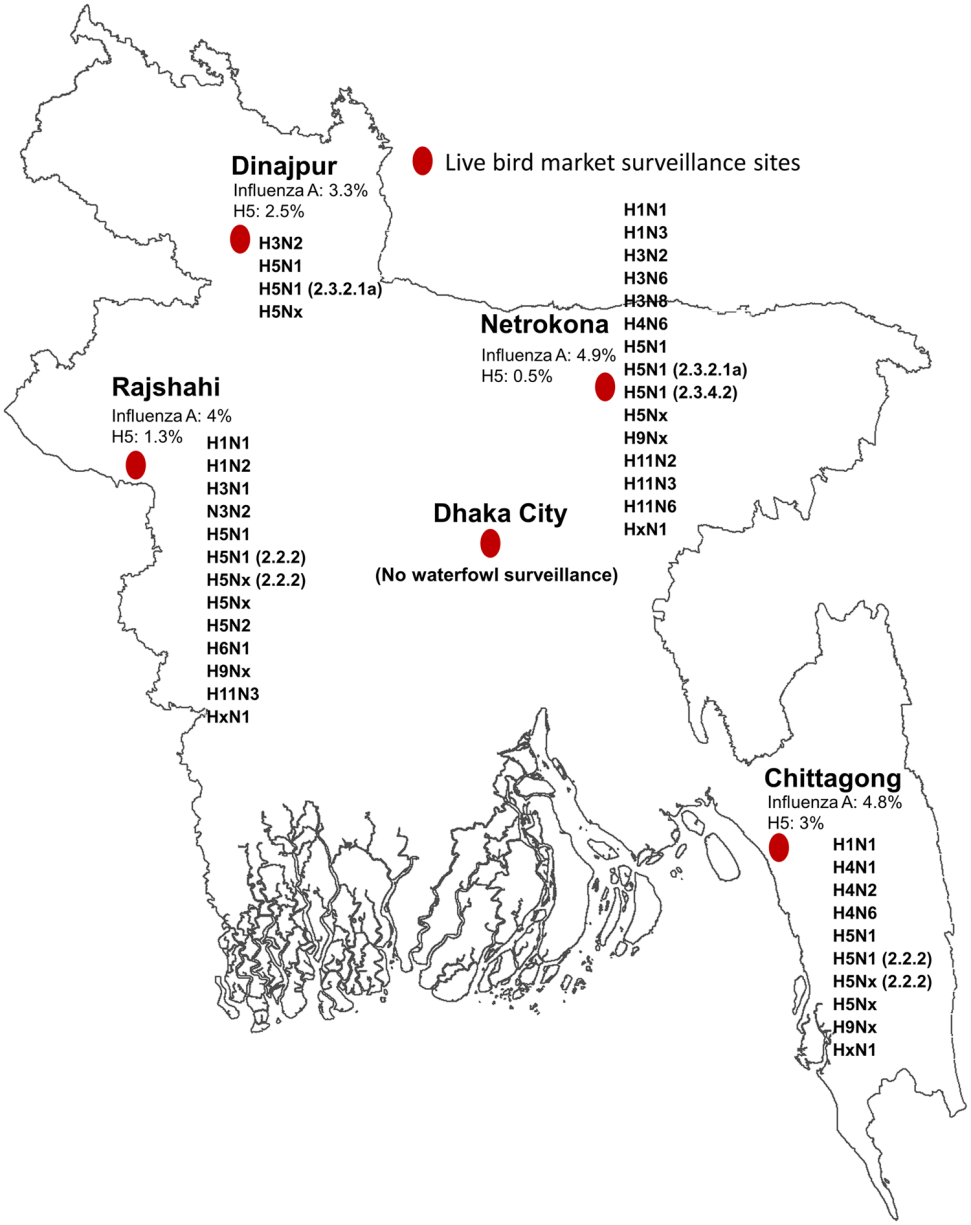
Location for the four rural live bird market avian influenza surveillance sites and Dhaka city, where the urban markets were sampled for influenza A viruses. Influenza A and H5 prevalence from the waterfowl samples and subtypes identified from waterfowl from each site were illustrated in the map of Bangladesh. The map was generated using ArcGIS version 10.4 (http://arcgis.com/).

**Figure 2:**
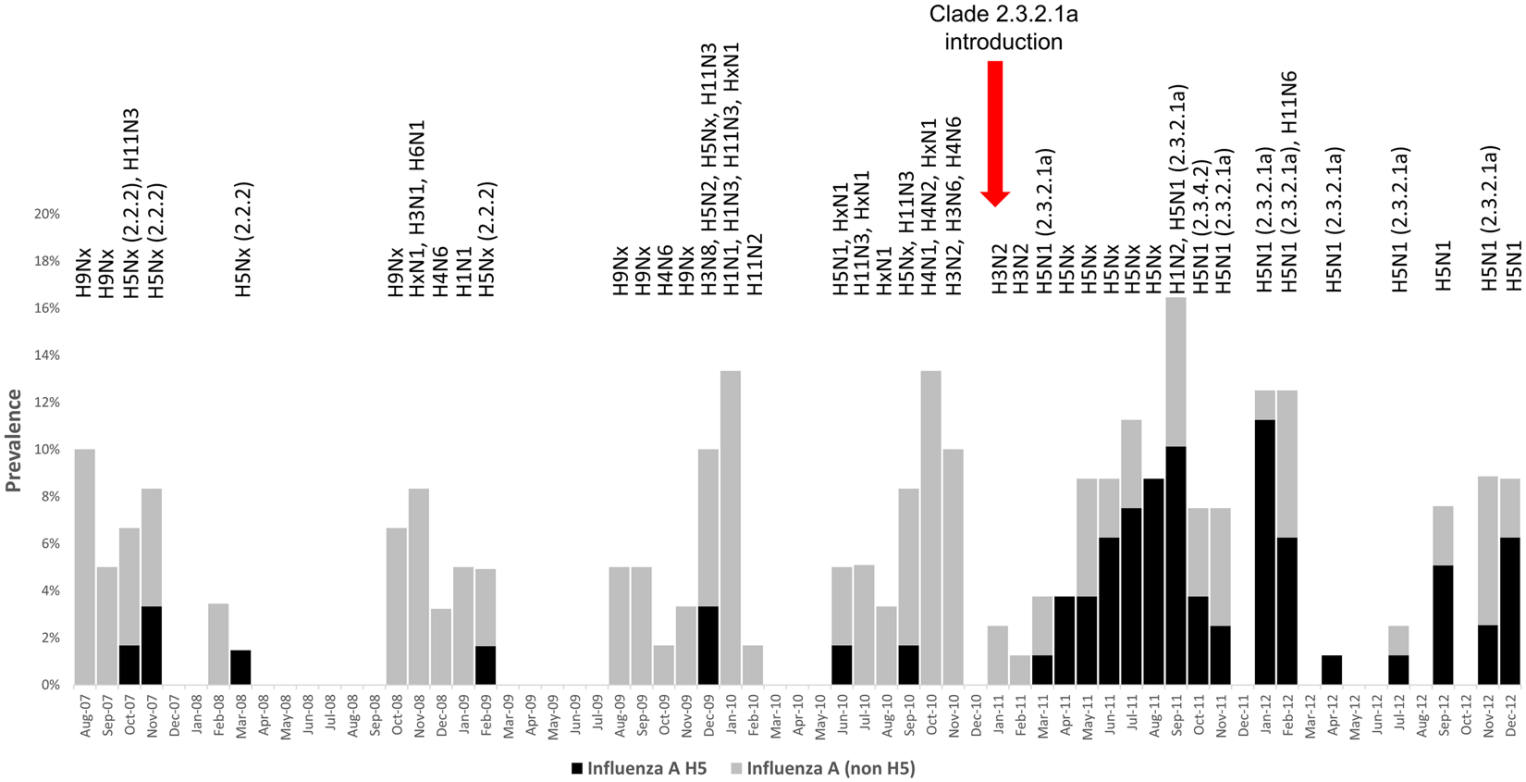
Prevalence of influenza A and H5 subtype of the viruses in the waterfowl samples from live bird markets in Bangladesh from August 2007 to December 2012. Subtypes and clades identified from each month’s sampling are presented on top of the bars.

Out of 4,308 waterfowl samples, 191 (4.4%) were positive for influenza A viruses in the live bird markets, including 74 (1.7%) that were influenza A/H5 positive. Influenza A virus prevalence varied from 3% [95% CI: 1.9–5.3%] to 5% [95% CI: 3.7–6.2%] depending on the market and ranged from 0.5% to 3% for influenza A/H5 viruses with the highest percentage observed at Chittagong (Fig. 1). Besides influenza A/H5 viruses, we also identified H1 (n = 6, 0.1%), H3 (n = 9, 0.2%), H4 (n = 8, 0.2%), H6 (n = 2, 0.001%), H9 (n = 9, 0.2%), and H11 (n = 10, 0.2%) among the influenza A positive waterfowl samples (Fig. 2, Appendix - Table 1). Hemagglutinin type for the remaining influenza A positive samples (38%) were not determined due to inability to propagate virus, amplify genomic material by PCR, and/or co-detection of Newcastle Disease virus RNA. Compared to the samples collected from backyard raised waterfowl, the small-scale operation raised waterfowl had more influenza A virus detections in their samples (3.6% vs. 5.4%, *P* < 0.01). However, the backyard raised waterfowl samples yielded more diverse hemagglutinin and neuraminidase subtypes (i.e. H1N1, H3N2, H3N6, H3N8, H4N1, H4N2, H4N6, H5N1, H5N2, H5Nx, H6N1, H9Nx, H11N2 and H11N3) compared to the viruses identified in the small scale raised waterfowl (i.e. H1N1, H1N3, H4N6 and H11N6). We did not identify evidence of co-infection by multiple influenza A subtypes through individual waterfowl sampling.

From 2007 to 2010, the majority of the influenza A virus detections in the live bird market occurred during the colder months (4.8% during October–March vs. 2.0% during April–September, *p* < 0.01). We also identified H5N1 in the live bird markets more often during the colder months (78% of the total H5 test positive samples were collected during October–March) of the year, though the difference was not statistically significant (0.2% during April–September vs. 0.6% in October–March, *p* = 0.2) (Fig. 2, Appendix-Figure 4, Appendix - Table 1). The surveillance samples collected from waterfowl in live bird markets were positive for three clades: 2.2.2, 2.3.2.1a, and 2.3.4.2 (Fig. 2). While clade 2.2.2 H5N1 viruses were exclusively detected during 2007–2010; clade 2.3.2.1a H5N1 viruses became the predominant clade detected by this surveillance following the introduction of the virus in early 2011 (Fig. 2, Appendix-Table 1).

The domesticated waterfowl sampled during October-March were more likely to test positive for influenza A viruses compared to the waterfowl sampled during April-September (odds ratio (OR) 1.5; 95% CI 1.1–2.0) (Table 2). Domesticated ducks were more likely to be infected with influenza A virus compared to geese (OR: 5.2, 95% CI: 2.1–12.7), and when infected, they were more likely to appear healthy at the time of sampling (OR: 3.4, 95% CI: 1.1–10.6) (Table 2). Birds raised in larger flocks with >20 birds and the birds raised in backyard flocks (vs. raised in small-scale operations) were less likely to be infected with influenza A/H5 viruses (Table 2).

**Table 2:** Multivariate analysis of the potential risk factors for identifying influenza A (N = 191) and influenza A/H5 (N = 74)in waterfowl in the live-bird markets, Bangladesh, 2007–2012.

Factors associated with influenza A infection in poultry flocks	Influenza A				Influenza A/H5			
	Bivariate		Multivariate		Bivariate		Multivariate	
	OR	95% CI	OR	95% CI	OR	95% CI	OR	95% CI
Influenza A seasonality (Oct–Mar = 1/Apr–Sep = 0)	**1.5**	**1.1–2.0**	**1.5**	**1.1–2.0**	0.9	0.6–1.4	—	—
Poultry flock size (≤20 = 0 vs. >20 = 1)	1.0	0.8–1.4	—	—	**0.4**	**0.2–0.7**	**0.2**	**0.1–0.5**
Dead birds in the week preceding sampling (≤2 = 0 vs. >2 = 1)	1.4	0.7–2.5	1.7	0.9–3.1	0.6	0.1–2.3	—	—
Percentages of dead birds in the week preceding sampling (<10% = 0 vs. ≥10% = 1)	1.5	0.8–2.7	—	—	0.7	0.2–2.7	—	—
Type of bird (ducks = 1 vs. geese = 0)	**5.2**	**2.1–12.7**	**5.3**	**2.2–12.8**	3.2	1.0–10.2	2.9	0.9–9.3
Age of bird (juvenile = 1 vs. adult = 0)	0.8	0.5–1.2	—	—	0.5	0.2–1.2	—	—
Health status (apparently healthy = 1 vs. sick = 0)	2.9	0.9–9.1	**3.4**	**1.1–10.6**	3.3	0.5–23.9	—	—
Type of farming (backyard = 1 vs. small scale = 0)	0.7	0.5–0.9	0.7	0.5–0.9	0.6	0.4–0.9	**0.5**	**0.3–0.9**

Note: OR – Odds Ratio; CI – Confidence Intervals; Boldface – Statistically significant at <0.05.

**Figure 3:**
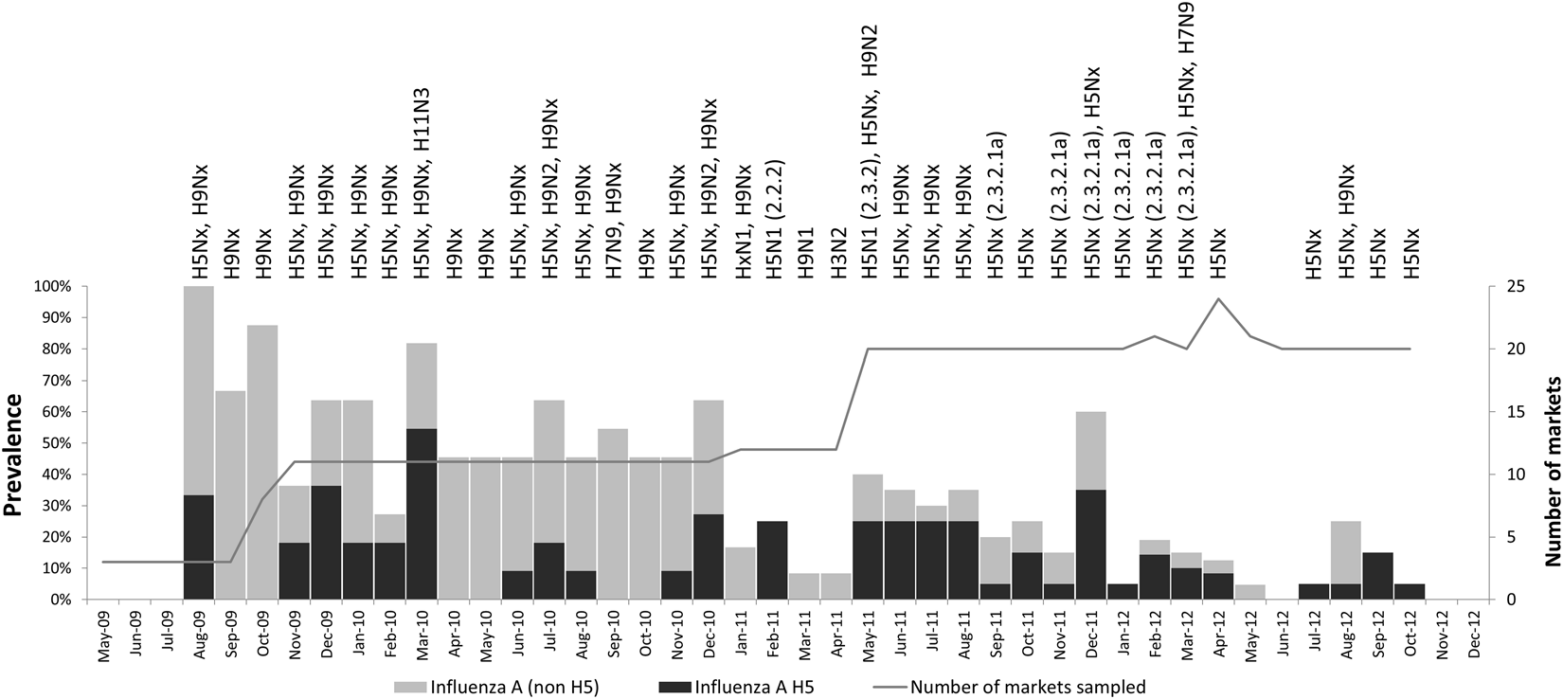
Prevalence of influenza A and H5 subtype of the viruses in the live bird market environmental samples in Bangladesh from May 2009 to December 2012. Subtypes and clades identified from each month’s sampling are presented on top of the bars.

We collected 590 pooled environmental samples from May 2009 through December 2012. One hundred and seventy three (29.3%) were positive for influenza A viruses and 74 (12.5%) were positive for influenza A/H5 virus by rRT-PCR (Fig. 3, Appendix Table 2). Besides H5 subtypes, H3 (n = 1, 0.2%), H7 (n = 2, 0.3%), H9 (n = 59, 10.0%), and H11 (n = 1, 0.2%) RNA were identified (Fig. 3). Among the pooled, influenza A positive environmental samples, 11 (1.9%) had RNA from multiple subtypes (Fig. 3, Appendix–Table 3).

A low pathogenic avian influenza (LPAI) A/H7N9 virus was identified in one environmental sample. BLAST analysis^19^ confirmed the virus to be of Eurasian lineage descent closely related to other LPAI H7 viruses circulating in the region but phylogenetically distant to the Chinese lineage H7N9 viruses^20^.

Pooled environmental swabs collected from both urban and rural markets tested positive for influenza A viruses by rRT-PCR. In the four rural live-bird markets, 17.6% (95% CI: 11.8–24.7%) of the 148 environmental specimens were rRT-PCR -positive for influenza A viruses, and 4% (95% CI: 3.4–14.8%) were positive for H5 virus. In the 16 urban markets in Dhaka, 33.3% (95% CI: 28.9–37.9%) of the 442 environmental specimens were rRT-PCR positive for influenza A viruses and 69 (15.6%) were positive H5N1 virus RNA. The environmental samples collected from the urban live bird markets were twice as likely to contain influenza A viruses (33.3% in urban vs. 17.6% in rural, p < 0.001) and five times more likely to contain influenza A/H5 viruses (15.6% in urban vs. 3.4% in rural, p < 0.001) than samples collected from rural live bird markets. Until the end of 2010, the majority (n = 20, 80%) of the H5 positive environmental samples in urban Dhaka markets were identified between October and March. Following the identification of clade 2.3.2.1a in March 2011, this seasonal trend was no longer apparent in 2011 and 2012 (15% in October–March vs. 10% in April–September, *p* = 0.09) (Fig. 3).

## Discussion

This surveillance complements previous study findings that showed that HPAI H5N1 and other influenza A viruses frequently circulated in apparently healthy waterfowl in the live-bird markets of Bangladesh^21–23^. We have previously published a manuscript (*Gerloff et al*. 2*016)*^20^ using low pathogenic influenza A virus sequence information from this LBM surveillance, a wild bird, and a community survey, and isolates from avian influenza outbreaks in Bangladesh from 2007 to 2013. The focus of the *Gerloff, et al. 2016* was to describe the ecology and evolution of the low pathogenicity avian influenza A viruses in Bangladesh from 2007 to 2013. However, this report builds on the prior reporting and focuses on the epidemiology of both high and low pathogenic avian influenza viruses identified in the LBMs in Bangladesh from 2007 to 2012. The magnitude and variety of influenza viruses in domestic poultry is an economic, food security and health concern^24–26^. The circulation of influenza A viruses throughout the year could be due to poultry-to-poultry transmission leading to entry into the poultry marketing chain and subsequent spread of disease at a national scale. It is also likely that substantial numbers of avian influenza infected poultry continuously enter the poultry marketing chain and serve as a source of infection for poultry kept in the urban live bird markets. Avian influenza circulation in the urban live bird markets could become sustained as urban live bird market closures and cleaning are not routinely practiced in Bangladesh^27^. The unsold birds and their excreta are likely in contact with uninfected birds brought in to the pool of susceptible birds in the markets and might maintain influenza viruses in the live bird markets.

The H5N1 virus isolates from Bangladesh collected from 2007 to 2009 were all clade 2.2.2 and showed very little (<5%) change in their genetic makeup^28^. This suggests that the influenza viruses, including H5N1 (clade 2.2.2) virus, have been maintained in reservoir hosts after their initial introduction into the country. Although a previous study reported very limited circulation of H5N1 in the live bird markets in Bangladesh^21^, we frequently identified the subtype in waterfowl brought into the markets. The majority of the H5N1 subtypes identified in our surveillance were circulating in domestic ducks, which in most cases, did not exhibit clinical illness. This finding is supported by several studies showing that ducks that carry influenza A viruses, including HPAI H5N1 virus, demonstrate very limited clinical signs or remain asymptomatic and shed the virus in feces and respiratory droplets^15,16,29,30^.

Poultry raised in backyards as free-ranging scavengers are known to be at risk of acquiring influenza viruses^31–33^ because of their increased contact with wild birds, other poultry and birds introduced or returning from live bird markets, and other environmental exposures. Poultry in larger-sized flocks (>20 birds) usually belonged to small-scale poultry operations; mostly consisting of free-ranging ducks raised for egg production that roam in rivers and other water bodies such as ponds, ditches, other wetlands and rice fields. These birds might acquire influenza A viruses through ingestion of water contaminated by other free ranging waterfowl^34^. Our multivariate analysis suggested that it was less likely to identify H5N1 infected waterfowl in the birds belonging to a larger flock size (>20) (Table 2). A study on similar (small scale) waterfowl operations examined egg yolk and found 24% of the samples had antibodies against H5N1, whereas fecal samples yielded very little evidence of active shedding of the virus^34^. Since most of the waterfowl from small scale operations were brought to the market after the end of the egg laying cycle, it is likely that we might have missed their active virus shedding period. Although the samples from small scale raised flocks had more influenza A viruses detections, the backyard raised flocks yielded more diverse subtypes, which might be explained by the diversity of exposure (both the environment and the commercial poultry marketing chain) to the backyard poultry enabling them to contract a variety of influenza A subtypes.

Since the introduction of the clade 2.3.2.1a in early 2011, there were reports of multiple outbreaks among poultry; many of the waterfowl exhibited clinical illness and mortality^35^. It is likely the clade 2.3.2.1a virus was either introduced to a completely naive waterfowl population or the new clade was highly virulent for the waterfowl population^35,36^. Our surveillance identified an influx of H5N1 detections right after the introduction of clade 2.3.2.1a in early 2011. This clade also likely replaced the previously circulating clade 2.2.2 (Figs 2 and 3)^37^. Clade 2.3.2.1a was considered to be one of the several invasive clades of H5N1 virus because of its ability to maintain efficient transmission within multiple species of poultry and wild birds and, subsequently, spread over a large geographic area^38^. Since the first reports of clade 2.3.2.1 H5N1 outbreaks in a common magpie in Hong Kong SAR in 2007, the virus continued to cause infection in domestic poultry and wild birds in multiple countries of Asia and Europe in recent years^39,40^. Previous studies have suggested that because clade 2.3.4.2 viruses were only found in domestic chickens that they might have been introduced through poultry trade. Since 2011, the viruses detected in Bangladesh were derived from clade 2.3.2.1, but not from 2.3.4.2 suggesting a lack of sustained circulation of this genetic group of H5N1 viruses in Bangladesh^41^.

Understanding seasonality of human and animal influenza viruses is important because the co-circulation of the viruses might increase the chances of co-infection among humans and animals leading to reassortment and emergence of novel influenza viruses capable of causing pandemics^42^. We identified more avian influenza A viruses occurring in colder months (October-March) (Appendix - Fig. 3). However, we were unable to identify any distinct seasonality for H5N1 virus occurrences (Appendix - Fig. 4). Reports from other countries in Asia observed a distinct seasonal pattern of H5N1 occurrence that peaked during autumn and winter months, demonstrating a strong tie between environmental factors and the occurrence of avian influenza in poultry^42,43^. In Bangladesh, it appears that anthropogenic factors, such as live bird markets and poultry marketing chains might contribute to the introduction of H5N1 infected poultry to the domestic poultry population throughout the year. Although the live bird market closure could be an intervention to interrupt transmission of avian influenza A viruses within the dynamic poultry population maintained within the marketing chain^44^, such interventions are seldom practiced in the urban live bird markets in Bangladesh. The circulation of H5N1 viruses throughout the year also leads to the possibility of co-circulation, co-infection and reassortment within the human and bird population^42^. Perhaps the currently ongoing avian influenza virus surveillance among live bird market poultry workers^45^ might help us better understand co-seasonality in humans and birds; if and when the virus co-infects, reassorts, acquires capacity to infect humans and turns out to be a public health concern.

Live-bird markets may play a key role in maintaining influenza viruses in poultry and its spread through market chain^46^. Identification of influenza viruses in market environmental samples highlights the persistence influenza viruses in the market environment throughout the year. Although rRT-PCR is unable to differentiate between presence of viable virus or only the viral nucleic acid, the steady identification in the market environment suggested sustained contamination of influenza A viruses through birds in the market chain. Through our surveillance and other studies in Asia, it is evident that multiple influenza A virus subtypes were simultaneously present in the live bird markets^1,8,10,47^ and there were chances that the birds brought to the market could be co-infected with multiple subtypes and clades. Co-infection of multiple subtypes of influenza A viruses within a host may result in the generation of a reassortant progeny as they are able to exchange their genetic material during the replication process^48^. The samples that we collected from individual birds did not identify more than one subtype co-infecting a waterfowl. While we did not identify an event of co-infection in the market waterfowl samples, a separate phylogenetic analysis of the H5N1 isolates identified from our surveillance demonstrated that the viruses reassorted within the same clade of H5N1, between different clades of H5N1, and between low pathogenic viruses (H9N2)^41^.

Influenza A viruse subtype H9N2 occur globally in poultry; a very small proportion of poultry-exposed humans have contracted the virus and the disease is often mild in nature^49^. Even though H9N2 has infected only a small number of humans, because of its widespread global distribution in poultry, poor immune response in humans, ability to reassort with other subtypes that may enhance poultry-to-human transmissibility or virulence the H9N2 subtype of influenza A virus has the potential to trigger a pandemic^49^. Occasionally human cases are identified through active influenza surveillance, as has occurred in Bangladesh during 2011–2015^50,51^, and many cases reported exposure to poultry and poultry products (e.g. raw meat) purchased from urban live bird markets^50^. It is likely that there are many undetected H9N2 cases in Bangladesh in humans exposed to infected poultry and live bird markets, but were never tested for influenza due to lack of severe illness or asymptomatic infections^52,53^.

Timely reporting of LBM avian influenza virus surveillance finding are important for public health preparedness. For this surveillance, we developed monthly-reports on the individual and cumulative monthly frequencies of influenza detections, including H5, H7, and H9 subtypes and disseminated to the following organizations: (a) Department of Livestock Services (DLS) of the Ministry of Fisheries and Livestock Services, Bangladesh, (b) Institute of Epidemiology, Disease Control and Research (IEDCR) of the Ministry of Health and Family Welfare, Bangladesh, (c) Local representative of the Food and Agricultural organization (FAO) of the United Nations, and (d) Influenza division of the Centers for Disease Control and Prevention (CDC), Atlanta, GA, USA and individuals associated with the live bird market surveillance and the local health emergency preparedness in Bangladesh. Data obtained from the results presented herein were included in assessment of zoonotic influenza virus vaccine strain selection performed by the WHO, from which a candidate vaccine virus, A/chicken/Bangladesh/11rs1984-30/2011, was recommended for development for pandemic preparedness purposes^54^, which was later developed by the CDC.

Detecting viral RNA from the live bird market environmental samples was particularly challenging. We applied rRT-PCR to detect nucleic acids of influenza A viruses from market environmental samples that might not be associated with viable viruses. rRT-PCR detections were followed by virus culture and other techniques including virus amplification, direct sequencing, and nucleotide ‘BLAST’ analysis to characterize the viruses. Since many of the environmental samples were positive for NDV RNA, these samples were destroyed to prevent possible isolation of velogenic NDV, a United States Department of Agriculture Select Agent Program policy that applies to the laboratories of the CDC Influenza Division. It was also difficult to culture viruses from environmental samples that contained bacteria that contaminated the eggs used to propagate the viruses. We therefore relied on multiplex PCR subtyping assays to detect virus in environmental samples. Samples with positively identified subtypes were then subjected to virus isolation and full length HA and NA sequence analysis.

Sixteen different HA/NA subtype combinations of avian influenza A viruses, including H5N1 and H9N2 viruses, were identified in backyard and small-scale poultry flocks brought to live bird markets in Bangladesh. LBMs may be high-risk sites for harboring these viruses. Market surveillance of waterfowl and environmental sampling might be a more efficient method to identify circulating highly pathogenic avian influenza virus strains when compared to waiting for passive reports of poultry outbreaks among backyard flocks and small-scale operations. Interventions to reduce market-based influenza contamination should be considered to reduce the introduction of influenza viruses in humans and domestic poultry as well as to reduce the chances of reassortment facilitated through the co-infection with multiple influenza viruses in the live bird markets^44,55^.

## Methods

### Selection of live-bird markets for poultry influenza surveillance

We conducted monthly surveillance for influenza viruses among domestic waterfowl in live-bird markets in Bangladesh from August 2007 to December 2012. In 2007, we selected three rural live-bird markets in three sub-districts of Netrokona, Rajshahi, and Chittagong districts as our rural surveillance sites based on their high poultry population density, presence of migratory waterfowl habitats, which was initially thought to have introduced the virus into the country, and relatively high density of domestic waterfowl^56,57^. On January 2011, we included another live bird market from a sub-district of Dinajpur district based on its similar characteristic. These four markets (Fig. 1) sold approximately 2000 poultry each on market days; between 2.5% and 20% of the birds sold were domestic waterfowl. In most cases, individual producers brought their birds for sale. These market days were fixed and varied from one market to another.

### Waterfowl sampling

A field team consisting of a veterinarian and a field assistant visited the markets once every month during a market day. The team approached owners of waterfowl to ask if they would be willing to let us sample their birds. Depending on the availability of birds, we sampled no more than four waterfowl per owner for a total of 20 waterfowl per market day. The team collected one of the following three types of samples from waterfowl: cloacal swab, swabs from freshly laid feces, or oropharyngeal swabs. Whenever we identified a waterfowl showing signs of respiratory disease, such as ocular or nasal discharge or swollen infraorbital sinuses, we collected an oropharyngeal swab. We rarely identified dead waterfowl in the live bird markets. When we did identify them, we collected a swab sample from the dead waterfowl following the procedure described earlier. The team followed WHO guidelines for sample collection and preservation^58^.

Along with the specimen collection from individual birds, the field team interviewed the waterfowl owners and collected information about the poultry farming system, types of birds reared, bird’s age, health status during specimen collection, flock size and history of mortality in the flock during the seven days preceding the sampling. We defined a waterfowl belonging to backyard poultry farming system as a bird housed at night but allowed to range freely during the day. We defined free-ranging small-scale farming as an operation where waterfowl were raised exclusively for meat and/or egg production; these were often nomadic in nature and were allowed for scavenge in the open fields and waterbodies during the day, and were kept in a temporary enclosure near the feeding grounds during night^59^.

### Live bird market environmental sampling for avian influenza viruses

The team also collected environmental samples once every month from the four rural markets and from 16 urban live bird markets in Dhaka, the capital and largest city in Bangladesh (Supplementary figure A-1). In the urban markets of Dhaka city, the dealers daily brought poultry from the peripheral live bird markets across the country. Once a month the team collected 10 swab samples from poultry cages, feed and water trays, and fecal materials from poultry stalls from different convenient sites in each market and pooled them into one environment sample from each market.

### Laboratory testing

We followed WHO recommended guidelines to maintain biosafety during collection, handling, preservation and laboratory analysis of the samples from waterfowl and live bird market environment^58^. At icddr,b the samples were maintained in a biosafety level 2 containment facility dedicated for animal sample testing and at CDC, all samples were maintained in biosafety level 3 containment, as required by the U.S. Department of Agriculture and the U.S. Federal Select Agent Program (http://www.cdc.gov/od/ohs/biosfty/bmbl5/bmbl5toc.htm).

We performed rRT-PCR for typing and subtyping of influenza A viruses using fluorescent Taqman probes at icddr,b and CDC, Atlanta. Total RNA was recovered from 100 µl of swab specimen collected in 2 ml viral transport medium using a commercial RNA extraction kit (RNeasy Mini kit, Qiagen, Valencia, CA, USA). The extracted RNA was screened for the presence of influenza A viruses by rRT-PCR using protocols recommended by Centers for Disease Control and Prevention (CDC)^60^. PCR master mix was prepared according to the manufacturer’s instruction (SuperScriptTM III Platinum(R) One-Step Quantitative RT-PCR System). Initial screening for influenza A virus, H5, or H9 was performed at icddr,b by real-time reverse transcription-polymerase chain reaction (rRT-PCR). An aliquot of each LBM sample that tested positive for influenza A virus by rRT-PCR was sent to CDC, and tested again initially against a panel of 5 different targets including influenza A virus, subtypes H5, H7, H9 and Newcastle disease virus^60^. Influenza A positive specimens negative for H5, H7 and H9 were further characterized by rRT-PCR using subtype specific primers to detect H1 and H3^41^. Samples with influenza A matrix gene ct values less than 30 were inoculated into 10–11 day old embryonated chicken eggs and allantoic fluid was harvested at either 24 (H5N1 virus) or 48 hours post-inoculation prior to testing by hemagglutination with turkey red blood cells to detected presence of virus^61^. The HA and NA genes of H5, H7 and H9-positive isolates were Sanger sequenced using subtype-specific primers. Influenza A positive (H5, H7 and H9 negative) samples that yielded virus isolates were subjected to a previously described multiplex PCR subtyping assay followed by HA and NA sequencing using subtype-specific primers^62^. We also tested the influenza A positive samples for the presence of Newcastle disease virus RNA, and excluded Newcastle disease virus positive samples from virus culture procedure^63^.

### Data analysis

We calculated descriptive statistics of the sampled waterfowl population and influenza A virus prevalence. We used the bivariate X^2^ tests of independence to assess the association between frequencies of the identifying influenza A virus and dichotomous clinical, demographic and farming variables. We estimated odds ratios (OR) with robust confidence intervals (CI) for the association between different potential exposures and harboring influenza A viruses and influenza A/H5 virus; these included occurrence of influenza A and influenza A/H5 viruses during October-March and April-September the colder and warmer months of the year^64^; poultry flock size above and below 20 birds (upper limit of the 95% confidence interval of the mean of the backyard raised poultry flocks); poultry mortality in flocks seven days preceding the sampling; species and age of the sampled waterfowl; and their health status during sampling. Variables that were statistically associated with influenza A or influenza A/H5 (p < 0.2) in the bivariate X^2^ tests of independence analysis, were entered into a multivariate logistic regression model to determine the association with the outcome (i.e. influenza A or influenza A/H5 test positive) and adjusted for confounding effects (i.e. age, bird type, raising pattern) and reported the adjusted odds ratio with 95% CI. Since the bird samples were from surrounding regions of the selected live bird markets, and sometimes, multiple samples were collected from a flock, we calculated robust confidence interval to guard against overestimation of the precision in estimates. We performed backward elimination and covariates with p < 0.05 were retained in the model. Using bivariate X^2^ tests, we assessed collinearity for the predictors retained in the final model. Finally, we performed *Hosmer-Lemeshow* X^*2*^ statistics^65^ for goodness-of-fit. We used Seasonal-Trend Decomposition method based on locally weighted regression, as a filtering procedure to decompose monthly influenza A and influenza A/H5 occurrence time series data in to trends, seasonal and reminder components^66^ to understand seasonality and trends of the influenza viruses in the poultry brought to the live bird markets. The figures with maps were generated using ArcGIS version 10.4 (http://arcgis.com/). Statistical analysis were performed using STATA version 13.1 (www.stata.com) and R version 3.3.1 (https://cran.r-project.org/).

### Ethical considerations

We developed a protocol (#2007-10) for surveillance and it was approved by ICDDR,B’s following institutional review boards: ‘*Research Review committee’, ‘Ethical Review Committee’, and ‘Animal Experimentation Ethics Committee’*. The surveillance components were implemented according to the protocol and all methods were performed in accordance with the relevant guidelines and regulations. During the sample collection, we described to the waterfowl owners the purpose of this surveillance, expected outcome, process of sampling, potential harm and benefits of being included in the study and obtained informed consent.

## Electronic supplementary material

Supplementary information
