# Supplementary material for: Avian influenza surveillance in domestic waterfowl and environment of live bird markets in Bangladesh, 2007–2012
Source: Sci Rep. 2018 Jun 20;8:9396. doi: 10.1038/s41598-018-27515-w (PMC6010472; doi:10.1038/s41598-018-27515-w)
Supplement: Supplementary file 1 — Supplementary information [file 41598_2018_27515_MOESM1_ESM.pdf]

APPENDIX

Avian influenza surveillance in domestic waterfowl and environment of live bird markets in Bangladesh, 2007-2012

Salah Uddin Khan, Emily S. Gurley, Nancy Gerloff, Md Z. Rahman, Natosha Simpson, Mustafizur Rahman, Najmul Haider, Sukanta Chowdhury, Amanda Balish, Rashid Uz Zaman, Sharifa Nasreen, Bidhan Chandra Das, Eduardo Azziz-Baumgartner, Katharine Sturm-Ramirez, C. Todd Davis, Ruben O Donis, Stephen P. Luby

CONTENTS

1. **Appendix - Figure 1** - Location for the four rural and an urban live bird markets for avian influenza surveillance in Bangladesh from 2007 to 2012. Duration of the site specific surveillance, surveillance type, and Influenza A and H5 prevalence from the waterfowl and environmental were illustrated in the map
2. **Appendix – Figure 2:** Location for the four rural and an urban live bird markets for avian influenza surveillance in Bangladesh from 2007 to 2012. Influenza A and H5 prevalence from the waterfowl samples and the locations of H5N1 outbreaks reported to World Organization of Animal Health, 2007-2013.
3. **STL analysis** – Additional information on methods
4. **Appendix - Figure 3:** The disintegrating results of influenza A virus occurrence by STL method. The filtering procedure was applied to decompose monthly influenza A occurrence time series data in to trends, seasonal and remainder components by using a weighted polynomial regression
5. **Appendix - Figure 4:** The disintegrating results of influenza A/H5N1 virus occurrence by STL method. The filtering procedure was applied to decompose monthly influenza A/H5N1 occurrence time series data in to trends, seasonal and remainder components by using a weighted polynomial regression
6. **Appendix - Table 1:** A list of the influenza A viruses (N=191) detected through live bird market waterfowl sampling in Bangladesh, 2007-2012
7. **Appendix – Table 2:** A list of the influenza A viruses detected through live bird market environmental sampling in Bangladesh, 2009-2012
8. **Appendix – Table 3:** A list of environmental surveillance sites (live bird markets), where we detected the co-presence of multiple influenza A subtypes in a pooled environmental swab sample from 2009 to 2012.
9. **References**

Appendix - Figure 1: Location for the four rural and an urban live bird markets for avian influenza surveillance in Bangladesh from 2007 to 2012. Duration of the site specific surveillance, surveillance type, and Influenza A and H5 prevalence from the waterfowl and environmental were illustrated in the map. The map was generated using ArcGIS version 10.4 (http://arcgis.com/).

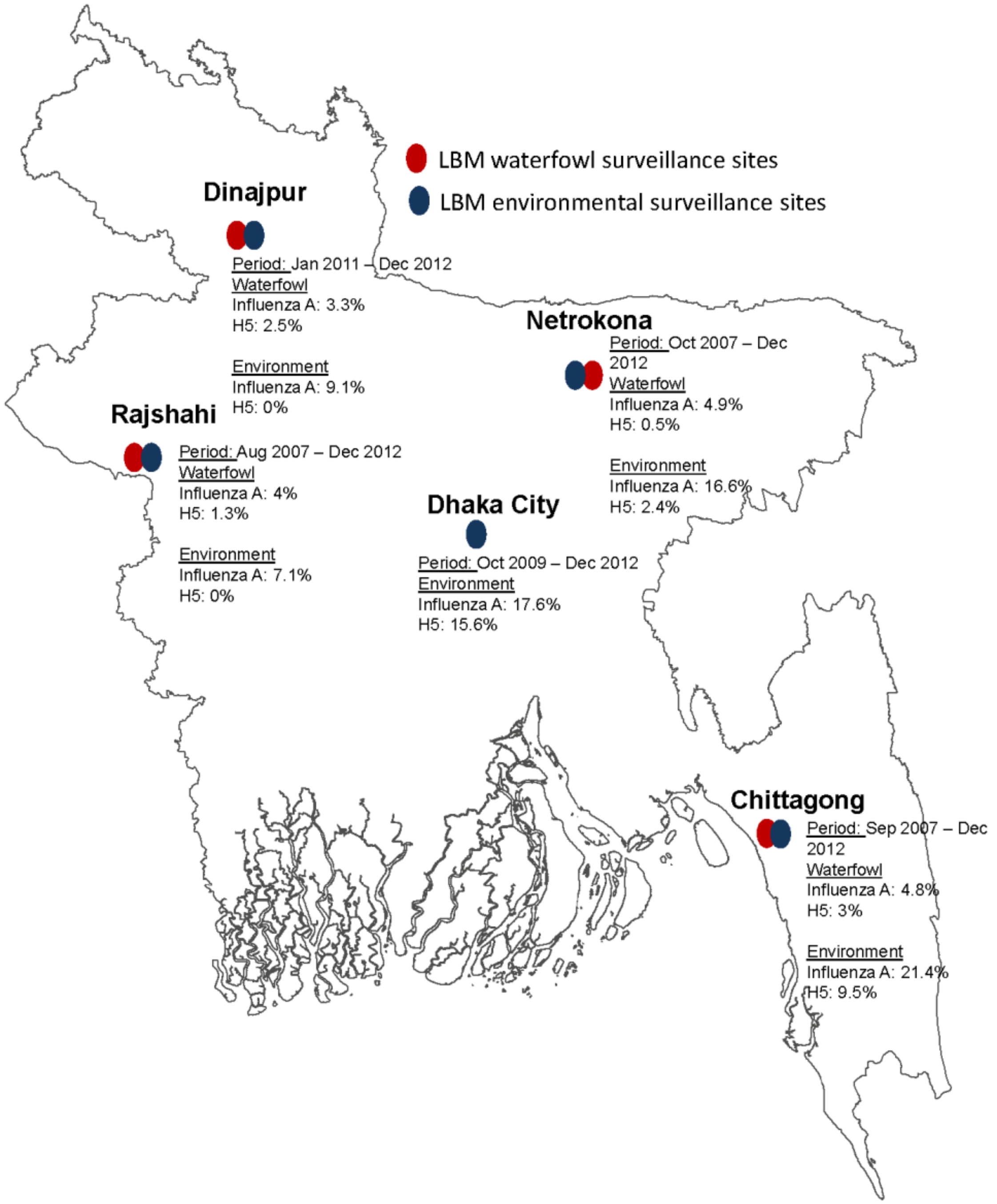

Appendix – Figure 2: Location for the four rural and an urban live bird markets for avian influenza surveillance in Bangladesh from 2007 to 2012. Influenza A and H5 prevalence from the waterfowl samples and the locations of H5N1 outbreaks reported to World Organization of Animal Health, 2007-2013. The map was generated using ArcGIS version 10.4 (http://arcgis.com/).

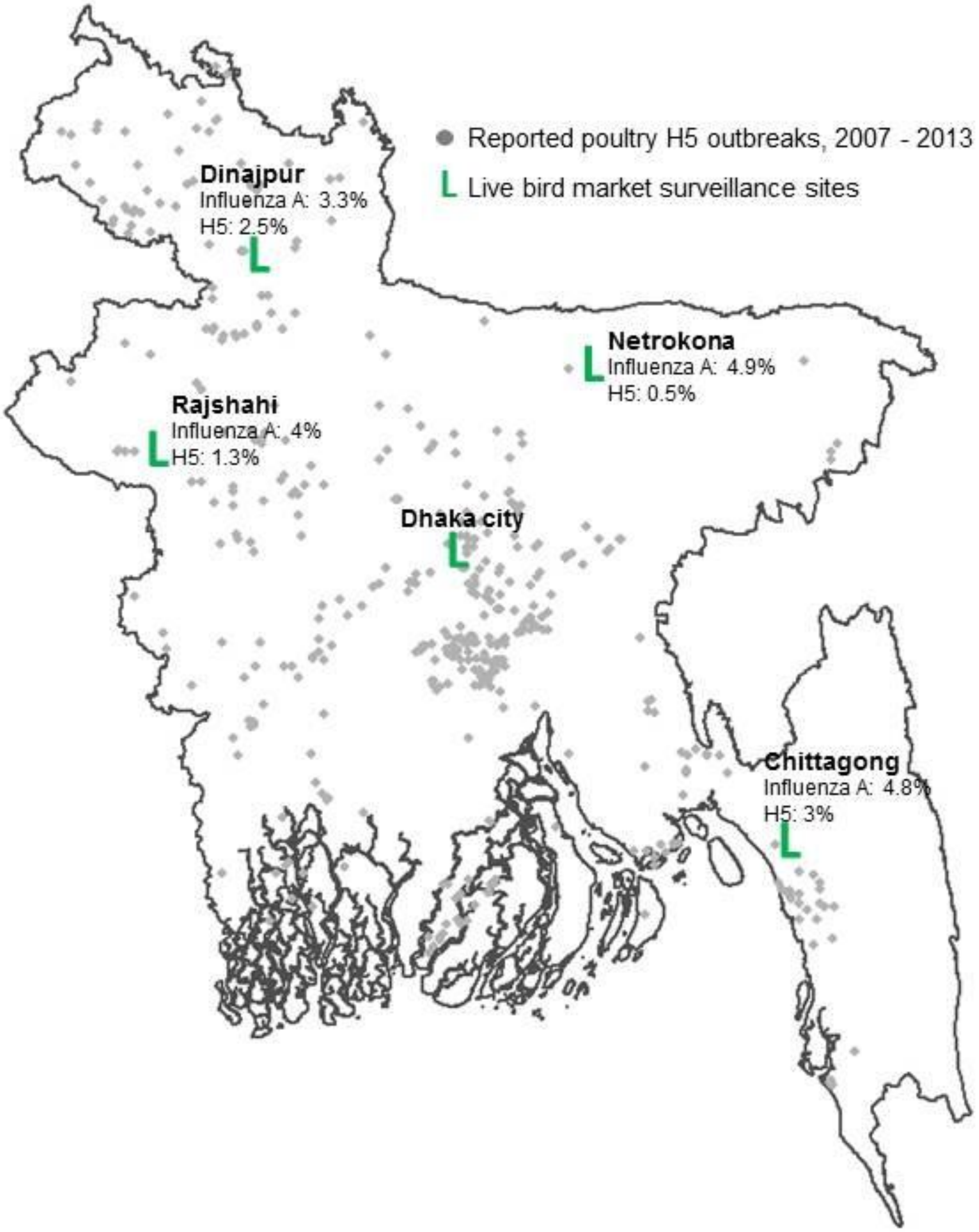

STL Analysis

We applied Seasonal-Trend Decomposition method based on locally weighted regression, also known as STL as a filtering procedure to decompose monthly influenza A and influenza A/H5 occurrence time series data in to trends, seasonal and remainder components by using a weighted polynomial regression [1, 2] to understand seasonality and trends of the influenza viruses in the poultry brought to the live bird markets.

$$Y_i = T_i + S_i + R_i$$

The equation above describes the components of the STL model, where Y_i is the element of the original time series (e.g. influenza A and H5 monthly detection data), T_i is the element for the trend variation which can be describes as low frequency variation in data along with nonstationary, consistent changes in level. S_i represents the seasonal component consisting variations in data within each seasonal frequency, here the length of each cycle is 1 year. The remainder (R_i) component consists of the data not explained by the trend and the seasonality. The gray bar at the right hand side of each category of the graph (Figure 3, Appendix: Figure 2) allows relative comparison of the magnitude of each components.

Appendix - Figure 3: The disintegrating results of influenza A virus detection by Seasonal-Trend Decomposition method. The filtering procedure was applied to decompose monthly influenza A detection time series data into trends, seasonal and remainder components by using a weighted polynomial regression. The Y-axes represents the periodic seasonal pattern of influenza A detection in the live bird markets and the expected trends.

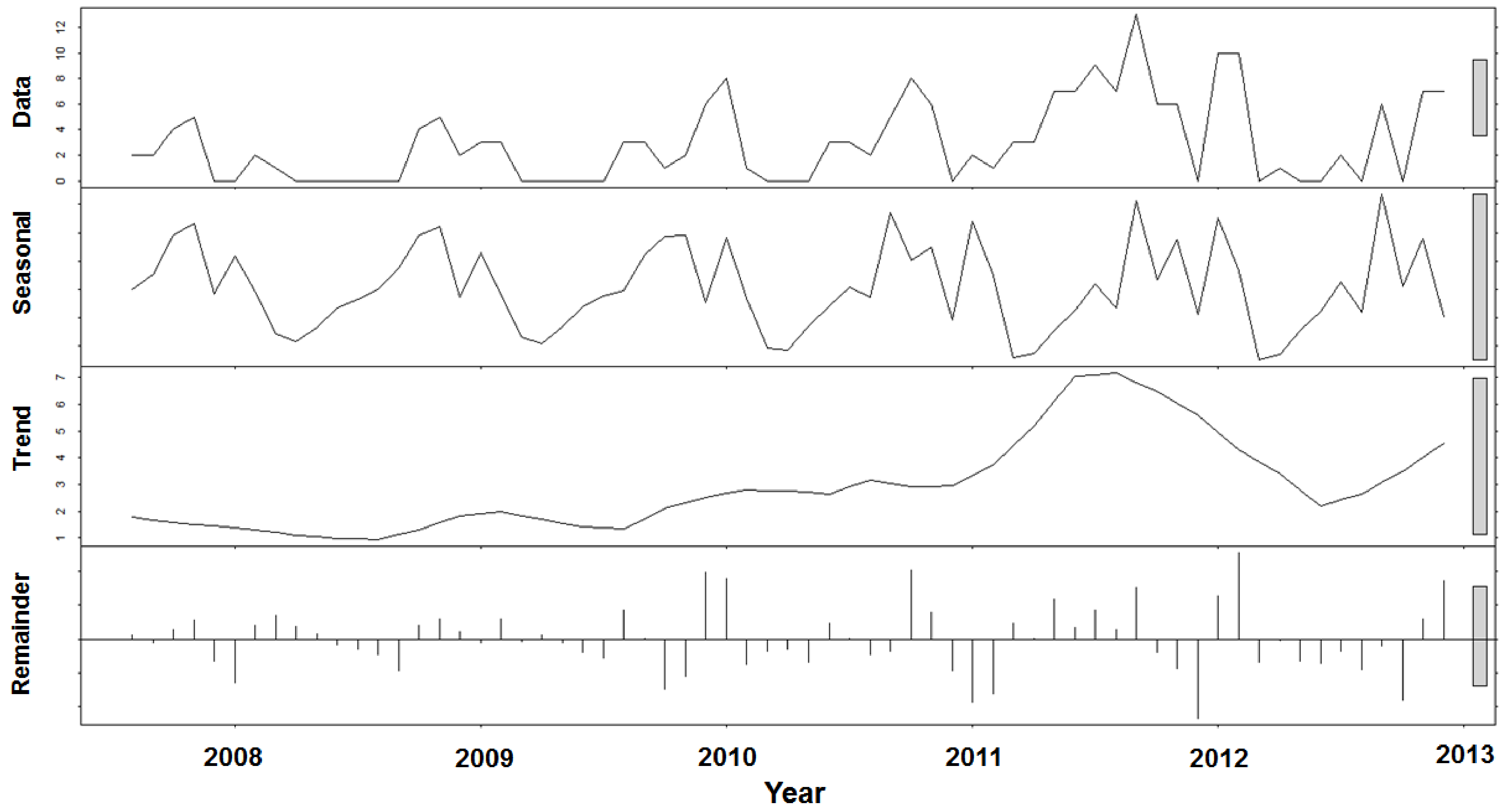

Appendix - Figure 4: The disintegrating results of influenza A/H5N1 virus detection by Seasonal-Trend Decomposition method. The filtering procedure was applied to decompose monthly influenza A/H5N1 detection time series data in to trends, seasonal and remainder components by using a weighted polynomial regression. The Y-axes represents the periodic seasonal pattern of influenza A/H5N1 detection in the live bird markets and the expected trends.

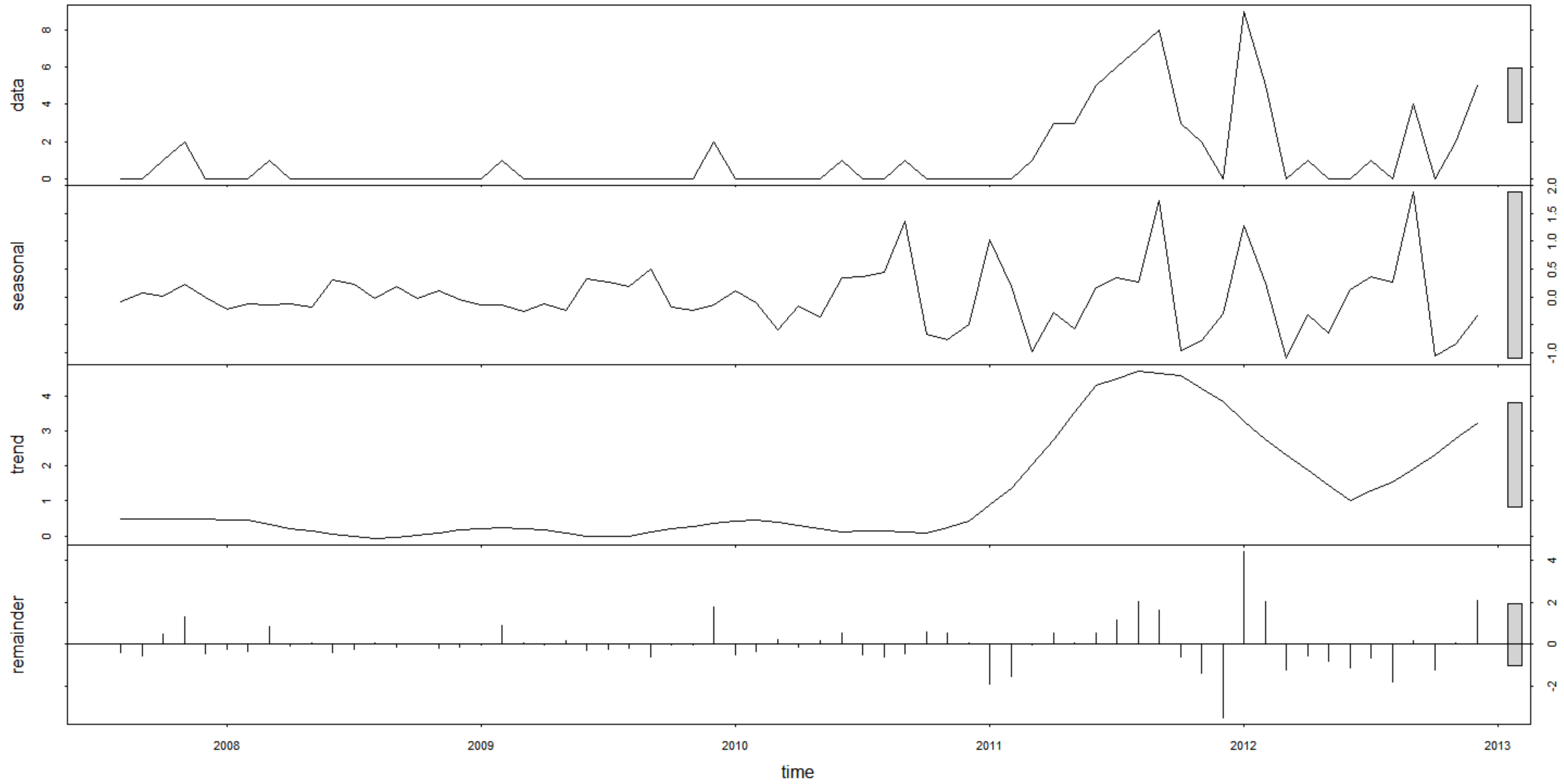

Appendix - Table 1: A list of the influenza A viruses (N=191) detected through live bird market waterfowl sampling in Bangladesh, 2007-2012.

Year	Month	Sampled host	Sample type	Health Status	Influenza A subtype(clade)	Number of test positive(s)
2007	August	Duck	Cloacal swab	Apparently Healthy	Undetermined	1
		Duck	Cloacal swab	Apparently Healthy	H9Nx	1
	September	Duck	Cloacal swab	Apparently Healthy	Undetermined	1
		Duck	Cloacal swab	Apparently Healthy	H9Nx	1
	October	Duck	Cloacal swab	Clinically ill	H5Nx(2.2.2)	1
		Duck	Cloacal swab	Apparently Healthy	H11N3	2
		Duck	Cloacal swab	Apparently Healthy	Undetermined	1
	November	Duck	Cloacal swab	Apparently Healthy	H5Nx	1
		Duck	Cloacal swab	Apparently Healthy	H5Nx(2.2.2)	1
		Duck	Tracheal swab	Apparently Healthy	Undetermined	1
		Duck	Cloacal swab	Apparently Healthy	Undetermined	2
2008	February	Duck	Cloacal swab	Apparently Healthy	Undetermined	1
		Duck	Tracheal swab	Apparently Healthy	Undetermined	1
	March	Geese	Tracheal swab	Apparently Healthy	H5Nx(2.2.2)	1
	October	Geese	Cloacal swab	Apparently Healthy	H9Nx	2
		Duck	Cloacal swab	Apparently Healthy	Undetermined	1
		Duck	Fecal	Apparently Healthy	Undetermined	1
	November	Duck	Fecal	Apparently Healthy	HxN1	1
		Duck	Fecal	Clinically ill	H3N1	1
		Duck	Fecal	Apparently Healthy	Undetermined	1
		Duck	Fecal	Apparently Healthy	H6N1	1
		Duck	Cloacal swab	Apparently Healthy	H6N1	1
	December	Duck	Cloacal swab	Apparently Healthy	H4N6	1
		Duck	Cloacal swab	Apparently Healthy	Undetermined	1
2009	January	Duck	Fecal	Clinically ill	Undetermined	1
		Duck	Fecal	Apparently Healthy	Undetermined	1
		Duck	Fecal	Apparently Healthy	H1N1	1
	February	Duck	Fecal	Apparently Healthy	Undetermined	2
		Duck	Cloacal swab	Apparently Healthy	H5Nx(2.2.2)	1
	August	Duck	Cloacal swab	Apparently Healthy	H9Nx	1
		Duck	Cloacal swab	Apparently Healthy	Undetermined	2
	September	Duck	Cloacal swab	Apparently Healthy	H9Nx	2
		Duck	Cloacal swab	Apparently Healthy	Undetermined	1
	October	Duck	Cloacal swab	Apparently Healthy	H4N6	1
	November	Duck	Cloacal swab	Apparently Healthy	H9Nx	2
	December	Duck	Cloacal swab	Apparently Healthy	H3N8	3
		Duck	Cloacal swab	Apparently Healthy	H5N2	1
		Duck	Cloacal swab	Apparently Healthy	H5Nx	1
		Duck	Cloacal swab	Apparently Healthy	H11N3	1
2010	January	Duck	Cloacal swab	Apparently Healthy	H1N1	1
		Duck	Cloacal swab	Apparently Healthy	H1N3	1

		Duck	Cloacal swab	Apparently Healthy	H11N3	1
		Duck	Cloacal swab	Apparently Healthy	HxN1	1
		Duck	Cloacal swab	Apparently Healthy	Undetermined	4
	February	Duck	Fecal	Apparently Healthy	H11N2	1
	June	Duck	Cloacal swab	Apparently Healthy	H5N1	1
		Duck	Cloacal swab	Apparently Healthy	HxN1	1
		Duck	Cloacal swab	Apparently Healthy	Undetermined	1
	July	Duck	Cloacal swab	Apparently Healthy	H1N1	2
		Duck	Cloacal swab	Apparently Healthy	HxN1	1
	August	Duck	Cloacal swab	Apparently Healthy	HxN1	1
		Duck	Cloacal swab	Apparently Healthy	Undetermined	1
	September	Duck	Cloacal swab	Apparently Healthy	H5Nx	1
		Duck	Cloacal swab	Apparently Healthy	H11N3	4
	October	Duck	Cloacal swab	Apparently Healthy	H4N1	1
		Duck	Cloacal swab	Apparently Healthy	H4N2	2
		Duck	Cloacal swab	Apparently Healthy	H4N6	1
		Duck	Cloacal swab	Apparently Healthy	HxN1	2
		Duck	Cloacal swab	Apparently Healthy	Undetermined	2
	November	Duck	Cloacal swab	Apparently Healthy	H3N2	1
		Duck	Cloacal swab	Apparently Healthy	H3N6	2
		Duck	Cloacal swab	Apparently Healthy	H4N6	2
		Duck	Cloacal swab	Apparently Healthy	Undetermined	1
2011	January	Duck	Cloacal swab	Apparently Healthy	H3N2	1
		Duck	Cloacal swab	Apparently Healthy	Undetermined	1
	February	Duck	Cloacal swab	Apparently Healthy	H3N2	1
	March	Duck	Cloacal swab	Apparently Healthy	H5N1(2.3.2.1a)	1
		Duck	Cloacal swab	Apparently Healthy	Undetermined	2
	April	Duck	Cloacal swab	Apparently Healthy	H5Nx	3
	May	Duck	Cloacal swab	Apparently Healthy	H5Nx	2
		Duck	Fecal	Apparently Healthy	H5Nx	1
		Duck	Cloacal swab	Apparently Healthy	Undetermined	4
	June	Duck	Cloacal swab	Apparently Healthy	H5Nx	5
		Duck	Cloacal swab	Apparently Healthy	Undetermined	2
	July	Duck	Cloacal swab	Apparently Healthy	H5Nx	6
		Duck	Cloacal swab	Apparently Healthy	Undetermined	3
	August	Duck	Cloacal swab	Apparently Healthy	H5Nx	7
	September	Duck	Cloacal swab	Apparently Healthy	H1N2	1
	September	Duck	Cloacal swab	Apparently Healthy	H5N1(2.3.2.1a)	7
		Duck	Cloacal swab	Apparently Healthy	H5N1	1
		Duck	Cloacal swab	Apparently Healthy	Undetermined	4
	October	Duck	Cloacal swab	Apparently Healthy	H5N1(2.3.4.2)	2
		Geese	Cloacal swab	Apparently Healthy	H5N1(2.3.4.2)	1
		Duck	Cloacal swab	Apparently Healthy	Undetermined	3
	November	Duck	Cloacal swab	Apparently Healthy	H5N1(2.3.2.1a)	2
		Duck	Cloacal swab	Apparently Healthy	Undetermined	4
2012	January	Duck	Cloacal swab	Apparently Healthy	H5N1(2.3.2.1a)	6
		Geese	Cloacal swab	Apparently Healthy	H5N1(2.3.2.1a)	1

		Duck	Cloacal swab	Apparently Healthy	H5N1	3
		Duck	Cloacal swab	Apparently Healthy	Undetermined	1
	February	Duck	Cloacal swab	Apparently Healthy	H5N1(2.3.2.1a)	3
		Duck	Cloacal swab	Apparently Healthy	H5N1	1
		Duck	Cloacal swab	Apparently Healthy	H11N6	1
		Duck	Cloacal swab	Apparently Healthy	Undetermined	4
	April	Duck	Cloacal swab	Apparently Healthy	H5N1(2.3.2.1a)	1
	July	Duck	Cloacal swab	Apparently Healthy	H5N1(2.3.2.1a)	1
		Duck	Cloacal swab	Apparently Healthy	Undetermined	1
	September	Duck	Cloacal swab	Apparently Healthy	H5N1	4
		Duck	Cloacal swab	Apparently Healthy	Undetermined	2
	November	Duck	Cloacal swab	Apparently Healthy	H5N1(2.3.2.1a)	2
		Duck	Cloacal swab	Apparently Healthy	Undetermined	5
	December	Duck	Cloacal swab	Apparently Healthy	H5N1	5
		Duck	Cloacal swab	Apparently Healthy	Undetermined	2

Appendix – Table 2: A list of the influenza A viruses detected through live bird market environmental sampling in Bangladesh, 2009-2012. The number of test positives represents the aggregated number of subtypes detected in each month from the live bird market environmental sampling sites.

Year	Month	Subtype (clade)	# Test Positives	NDV co-presence
2009	Aug	H5Nx	1	-
		H9Nx	1	-
		Undetermined	1	-
	Sep	H9Nx	2	-
	Oct	H9Nx	5	-
		Undetermined	2	-
	Nov	H5Nx	2	-
		H9Nx	2	-
	Dec	H5Nx	4	-
		H9Nx	3	-
2010	Jan	H5Nx	2	-
		H9Nx	5	-
	Feb	H5Nx	2	-
		H9Nx	1	-
	Mar	H5Nx	6	-
		H9Nx	2	-
		H11N3	1	-
	Apr	H9Nx	5	-
	May	H9Nx	4	-
	Jun	H5Nx	1	-
		H9Nx	2	-
		Undetermined	2	-
	Jul	H5Nx	2	-
		H9N2	1	-
		H9Nx	2	-
		Undetermined	2	-
	Aug	H5Nx	1	-
		H9	4	-
	Sep	H7N9	1	-
		H9Nx	2	-
		Undetermined	3	-
	Oct	H9N2	1	-
		H9Nx	3	-
		Undetermined	1	-
	Nov	H5Nx	1	-
		H9Nx	3	-
		Undetermined	1	-
	Dec	H5Nx	4	-
		H9N2	1	-

		H9Nx	1	-
		Undetermined	1	-
2011	Jan	H9Nx	1	-
		HxN1	1	-
	Feb	H5N1(2.2.2)	3	Yes
	Mar	H9N1	1	-
	Apr	H3N2	1	-
	May	H5N1(2.3.2)	1	Yes
		H5Nx	4	Yes
		H9Nx	2	-
		Undetermined	1	-
	Jun	H5Nx	5	Yes
		H9Nx	1	-
		Undetermined	1	-
	Jul	H5Nx	5	-
		H9Nx	1	-
	Aug	H5Nx	5	Yes
		H9Nx	1	-
		Undetermined	1	-
	Sep	H5Nx(2.3.2.1a)	1	-
		Undetermined	3	-
	Oct	H5Nx	3	-
		Undetermined	2	-
	Nov	H5Nx	1	-
		Undetermined	2	-
	Dec	H5Nx(2.3.2.1a)	5	Yes
		H5Nx	2	-
		Undetermined	5	-
2012	Jan	H5Nx(2.3.2.1a)	1	Yes
	Feb	H5Nx(2.3.2.1a)	2	Yes
		H5Nx	1	-
		Undetermined	1	-
	Mar	H5Nx(2.3.2.1a)	1	Yes
		H5Nx	1	-
		H7N9	1	-
	Apr	H5Nx	2	-
		Undetermined	1	-
	May	Undetermined	1	-
	June	-	-	-
	July	H5Nx	1	-
	Aug	-	-	-
		H5Nx	1	-
		H9Nx	1	-
		Undetermined	3	Yes
	Sep	H5Nx	3	-

Oct	H5Nx	1	-

Appendix – Table 3: A list of environmental surveillance sites (live bird markets), where we detected the co-presence of multiple influenza A subtypes in pooled environmental swab samples from 2009 to 2012. We only detected influenza A H5Nx and H9Nx virus co-presence, all of which were during 2011. We also detected co-presence of Newcastle disease virus RNA in all these samples.

Year	Month	Live bird market name
2011	May	Shantinagar Kacha Bazar, Dhaka city
		Mohammadpur Townhall City Corporation Market, Dhaka city
	Jun	Mohammadpur Townhall City Corporation Market, Dhaka city
		Kaptanbazar Market, Dhaka city
	Jul	Mohammadpur Townhall City Corporation Market, Dhaka city
		Nawab Yusuf Ali Kacha Bazar, Dhaka city
		Hazaribag Kacha Bazar, Dhaka city
		Mohakhali Kancha Market, Dhaka city
	Aug	Moulovibazar Kacha Bazar, Dhaka city
		Rampura kacha market, Dhaka city
		Mohammadpur Townhall City Corporation Market, Dhaka city

REFERENCES

1. Cleveland, R.B., W.S. Cleveland, and J.E. McRae, *STL: A seasonal-trend decomposition procedure based on loess.* J Official Stat, 1990. **6**: p. 3-73.
2. Ke, G., et al., *Epidemiological analysis of hemorrhagic fever with renal syndrome in China with the seasonal-trend decomposition method and the exponential smoothing model.* Sci Rep, 2016. **6**: p. 39350.
